# Impact of Fundamental Diseases on Patients With COVID-19

**DOI:** 10.1017/dmp.2020.139

**Published:** 2020-05-07

**Authors:** Yiguang Chen, Tianhua Li, Yongyi Ye, Yongjian Chen, Jun Pan

**Affiliations:** Department of Neurosurgery, Nanfang Hospital, Southern Medical University, Guangzhou, Guangdong, China; Department of Medical Oncology and Guangdong Key Laboratory of Liver Disease, the Third Affiliated Hospital of Sun Yat-sen University, Guangzhou, Guangdong, China; Guangdong Medical University, ZhanJiang, Guangdong, China

**Keywords:** COVID-19, SARS-CoV-2, The elderly

## Abstract

**Objectives:**

In December 2019, a new type of coronavirus, called severe acute respiratory syndrome coronavirus 2 (SARS-CoV-2), appeared in Wuhan, China. Serious outbreaks of coronavirus disease 2019 (COVID-19), related to the SARS-CoV-2 virus, have occurred throughout China and the world. Therefore, we intend to shed light on its potential clinical and epidemiological characteristics.

**Methods:**

In this retrospective study, we included 50 confirmed fatal cases of SARS-CoV-2 reported on Chinese official media networks from January 16, 2020, to February 5, 2020. All the cases were confirmed by local qualified medical and health institutions. Specific information has been released through official channels. According to the contents of the reports, we recorded in detail the gender, age, first symptom date, death date, primary symptoms, chronic fundamental diseases, and other data of the patients, and carried out analyses and discussion.

**Results:**

In total, 50 fatal cases were reported: median age was 70 y old, and males were 2.33 times more likely to die than females. The median number of days from the first symptom to death was 13, and that length of time tended to be shorter among people aged 65 and older compared with those younger than 65 (12 days vs 17 days; *P* = 0.046). Therefore, the older patients had fewer number of days from the first symptom to death (r = -0.40; *P* = 0.012).

**Conclusions:**

In our study, we found that most of the deaths were elderly men with chronic fundamental diseases, and their COVID-19 progression to death time was shorter. At the same time, we demonstrated that older men are more likely to become infected with COVID-19, and the risk of death is positively correlated with age.

Since December 2019, an infectious disease, caused by the severe acute respiratory syndrome coronavirus 2 (SARS-CoV-2),^1^ has spread from Wuhan to other parts of China, also to other countries.^2^ On February 11, 2020, the World Health Organization (WHO) named the disease caused by SARS-CoV-2 as “coronavirus disease 2019” or “COVID-19.”^3^ In the early stages of the disease, patients show severe acute respiratory infection symptoms. Some patients rapidly develop acute respiratory distress syndrome (ARDS), acute respiratory failure, and even die.^4^ Although the Chinese government has blockaded Wuhan, the number of infections and deaths continues to rise. Doctor Wenliang Li, named “Sentinel” by the Chinese people, also unfortunately died of this infectious disease. The statistics are not only numbers, they also represent suffering lives.

Like severe acute respiratory syndrome (SARS) and middle east respiratory syndrome (MERS), COVID-19 also is characterized by flu-like symptoms, including fever, cough, and anhelation, and has the possibility of transmission from animals to humans.^2,5^ At the time of this writing, compared with the other two viruses, the SARS-CoV-2 has relatively mild symptoms and low mortality, but is more contagious.^6^

We have collected relevant information about 50 deaths confirmed to have been caused by COVID-19 and made some new analyses to improve the understanding of the disease.

## METHODS

### Data Sources and Searches

In this retrospective study, we collected data from official Chinese websites and news sources and also included demographic information about each death from January 16, 2020, to February 5, 2020. We acquired data published on January 16, 2020^7^; January 21, 2020^8^; January 23, 2020^9^; January 25, 2020^10^; January 27, 2020^11^; January 30, 2020^12^; February 2, 2020^13^; February 5, 2020.^14^ Our research has passed the review of the ethics committee of Southern Medical University.

### Statistics

We apply median values to describe the difference between two groups, and correlation coefficients to describe the correlation between two indicators. Wilcoxon tests were performed for univariate analyses. Excel was used for data input and collation, and R software version 3.6.0 and MedCalc 15.0 were used for descriptive analyses and statistical analyses. A *P* < 0.05 was considered to be statistically significant.

## RESULTS

### Basic Characteristics of Cases

**FIGURE 1:**
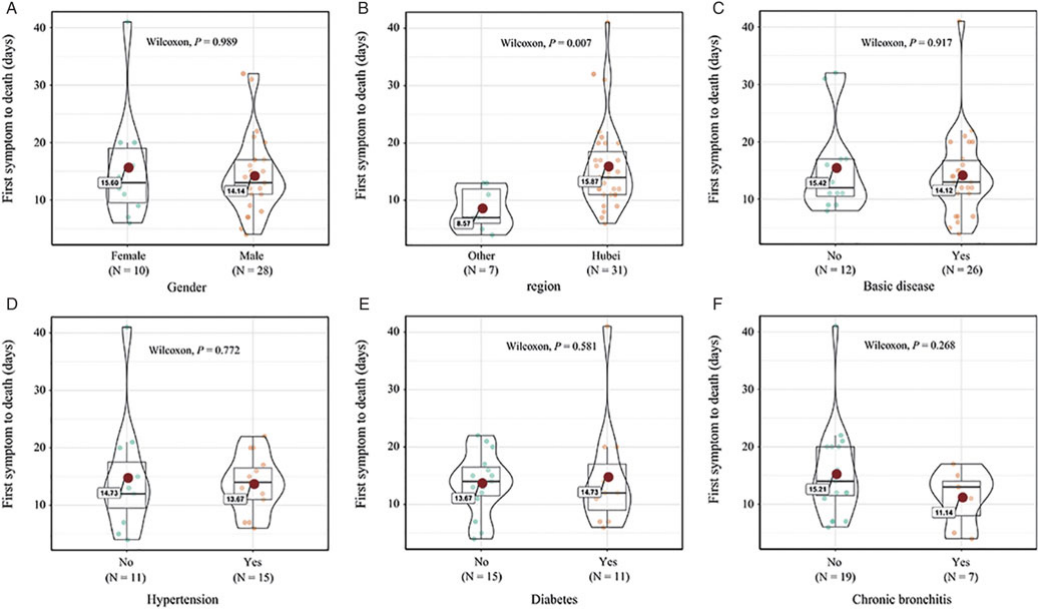
A-F, Comparison of the Influence of Different Factors on the Time of First Symptom to Death (Days). Violin plots for (A) gender, (B) region, (C) fundamental disease, (D) hypertension, (E) diabetes, and (F) chronic bronchitis.

From January 9, 2020, to February 4, 2020, a total of 50 deaths caused by infection with the new coronavirus pneumonia were reported: 35 males and 15 females. The gender ratio of male to female was 2.33:1. The age span of the patients was between 36 and 89 y old; the average age was 70.2 y old; and the median age was 70 y old. The elderly account for the majority of the deaths: 35 patients were older than 65 y old, accounting for 70% of the total cases. Hubei has the highest disease density. Thirty-nine cases were reported in Hubei, accounting for 78%; 2 cases were reported in Henan; 2 cases were reported in Chongqing; 1 case was reported in Beijing; 1 case was reported in Shanghai; 1 case was reported in Hebei, 1 case was reported in Hainan; 1 case was reported in Sichuan; 1 case was reported in Hong Kong; and 1 case was reported in Philippines (Table 1 and Figure 1A,B).

**TABLE 1:** Baseline Characteristics of Death Cases

Value	N = 50
Gender (%)	
Male	35 (70.0)
Female	15 (30.0)
Age (median (IQR))	70 (65, 81)
Mean (SD)	70.18 (13.37)
<65	15 (30.0)
>65	35 (70.0)
Basic disease (%)	
Yes	33 (66.0)
No	17 (34.0)
Fever (%)	
Yes	37 (82.2)
No	8 (17.8)
Cough (%)	
Yes	25 (55.6)
No	20 (44.6)
Dyspnea (%)	
Yes	28 (62.2)
No	17 (37.8)
Myalgia or fatigue (%)	
Yes	3 (6.7)
No	42 (93.3)
Digestive symptoms (%)	
Yes	12 (26.7)
No	33 (73.3)
Lethargy or coma(%)	
Yes	5 (11.1)
No	40 (88.9)
Hypertension (%)	
Yes	17 (51.5)
No	16 (48.5)
Diabetes (%)	
Yes	13 (39.4)
No	20 (60.6)
Chronic bronchitis (%)	
Yes	7 (21.2)
No	26 (78.8)
The days of first symptom to death (median (IQR))	13 (11, 17)
Region (%)	
Hubei	39 (78.0)
Other	11 (22.0)

### Analysis of Clinical Characteristics

**FIGURE 2:**
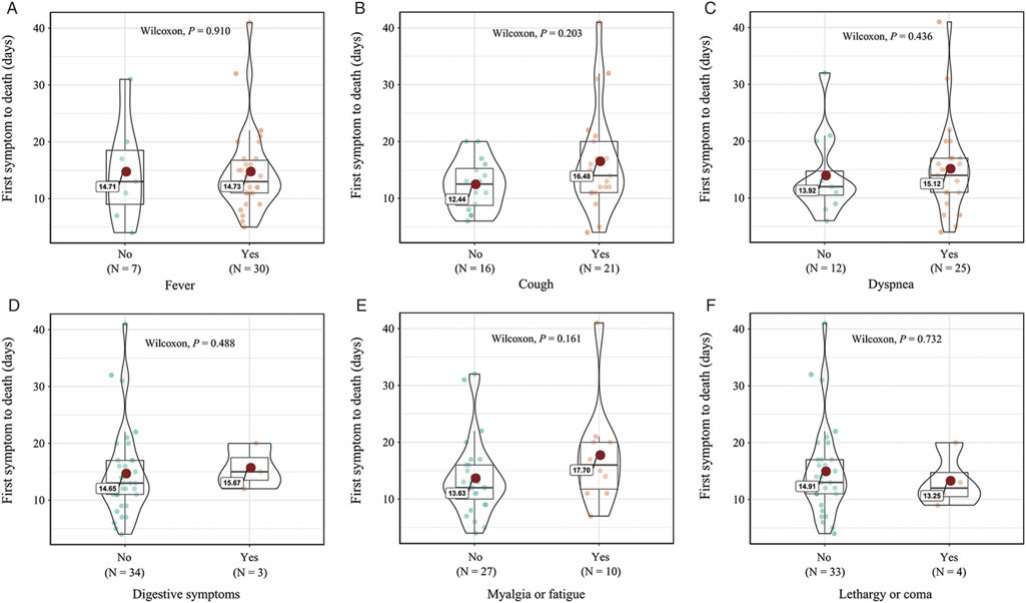
A-F, Comparison of the Influence of Clinical Features on the Time of First Symptom to Death (Days). Violin plots for (A) fever, (B) cough, (C) dyspnea, (D) digestive symptoms, (E) myalgia or fatigue, and (F) lethargy or coma.

Symptom information of 45 cases was collected for review. The period of time from first symptom to death ranged between 4 and 41 days; the average time from morbidity to death was 14.5 days, with a median of 13 days. Among the cases, fever, cough, and dyspnea were the main symptoms, accounting for 82.2%, 55.6%, and 62.2%, respectively. Twelve patients had myalgia or fatigue, accounting for 26.7%. Three patients had digestive symptoms, accounting for 6.7%. Five patients had lethargy or coma, accounting for 11.1% (Table 1 and Figure 2). Thirty-seven patients had fever ≥ 38°C. Most of the patients were admitted to the intensive care unit in late stages of disease and showed critical clinical manifestations, such as respiratory failure and ARDS. Thirty-three cases also had other chronic fundamental diseases, accounting for 66.0% of the deaths, hypertension, chronic bronchitis, and diabetes being the most frequent, which accounted for 51.5%, 39.4%, and 21.2% of deaths, respectively (Table 1; Figure 1C-F).

### Univariate Analysis

**FIGURE 3:**
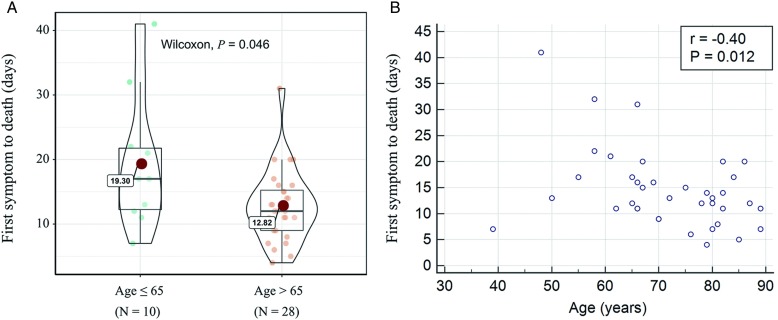
A,B, Influence of Age on the Time of First Symptom to Death (Days). A, Violin plots for age. B, Correlation between age and the time of first symptom to death (days).

Univariate analysis was performed on gender, age, region, date of first symptom, and fundamental disease. We found that age was a factor that affects the average number of days from symptom onset until death. The time from the first symptom to death in the elderly older than 65 y old was shorter than that in other groups. The median number of days from the first symptom to death was 13, ranging from 4 days to 41 days. It tended to be shorter among people aged 65 and older (12 [interquartile range {IQR} = 6.75] days) than those younger than 65 (17 [IQR = 12.75] days; *P* = 0.046) (Table 2; Figure 3A).

**TABLE 2:** Univariate Analysis

Parameter	Median (IQR)		Kruskal-Wallis Test *P*-Value
Gender (male vs female)	13 (11, 17)	13 (8.5, 20)	0.987
Age (<65 vs >65)	17 (11.75, 24.5)	12 (9, 15.75)	0.044
Region (Hubei vs other)	14 (11, 20)	7 (5, 13)	0.005
Fever (yes vs no)	13 (11, 17)	13 (7, 20)	0.410
Cough (yes vs no)	14 (11, 20)	12.5 (8.25, 15.75)	0.183
Dyspnea (yes vs no)	14 (11,17)	12 (9.5, 18.25)	0.313
Digestive symptoms (yes vs no)	15 (12, NA)	13 (10.5, 17)	0.319
Myalgia or fatigue (yes vs no)	16 (11,20.25)	12 (9, 16)	0.155
Lethargy or coma (yes vs no)	12 (9.5, 18.25)	13 (11, 17)	0.392
Basic disease (yes vs no)	13 (10, 17.75)	12 (9.5, 17)	0.914
Hypertension (yes vs no)	14 (11, 17)	12 (7, 20)	0.936
Diabetes (yes vs no)	12 (7, 20)	14 (11, 17)	0.876
Chronic bronchitis (yes vs no)	13 (5, 15)	14 (11, 20)	0.555

### Correlation Analysis

Scatter plot and correlation analyses on age and the number of days from the first symptom to death were performed. We found that there was a correlation between age and average number of days until death, and the difference was statistically significant. The correlation coefficient was −0.40, and the *P* value was 0.012 (Figure 3B).

## DISCUSSION

At the time of this writing, the outbreak of COVID-19 in Wuhan, other parts of China, and 24 other countries around the world has been occurring for nearly 2 months. According to the official announcement on February 10, 2020, 0:00 am, by the National Health Commission of the People’s Republic of China,^15^ the number of confirmed cases of COVID-19 is 37,213, the death toll is 815, the current mortality is 2.2%.

Research to study this virus and the disease is ongoing. Peng Zhou et al.^16^ found that 79.5% of the sequence of SARS-CoV-2 is identical to that of SARS-CoV, and the virus has high homology with the bat coronavirus at the whole-genome level (96%). It is also proven that SARS-CoV-2 infects the body through the ACE2 receptor on cells, the same mechanism of infection as SARS-CoV. In clinical reports, De Chang et al.,^17^ studying data from 13 patients, reported that the median age of patients was 34 y old. Most of the patients had a fever before admission, and the main symptoms were cough (46.3%) and upper airway congestion (61.5%).

Prof Chen et al.^5^ considered that, among the 99 patients they studied, approximately half had a history of contact with the Wuhan South China Seafood Market, and most of the patients were male, with an average age of 55.5 y (SD, 13.1), and 50 (51%) patients had another chronic disease before they acquired COVID-19. At present, we find that, although there are many published reports, few studies focused on death from COVID-19. One such study, by Wang et al.,^18^ reported that deaths from SARS-CoV-2 pneumonia were mainly among the elderly. To learn more, we retrospectively collected data on 50 deaths caused by COVID-19 and carried out this analysis.

In this study, we observed that more males died than females, which was consistent with previous reports of MERS-CoV and SARS-CoV.^19,20^ In addition, most patients had different types of underlying chronic fundamental diseases, mainly cardiovascular diseases and chronic bronchitis, as well as diabetes, before contracting COVID-19, which is similar to the patients who contracted MERS.^21-23^ The main symptoms of fever, cough, respiratory discomfort, and a small proportion of gastrointestinal symptoms, were consistent with symptoms in patients with SARS and MERS.^22,24^ Our results showed that the clinical features of COVID-19 were similar to those of the previous epidemic’s coronavirus pneumonia and that it is more likely to infect older men with chronic comorbidities, as a result of the weak immune function of these patients and stronger immune response in females caused by the additional X chromosome.^22,23,25^

At the time of this writing, compared with SARS (9.2%)^26^ and MERS (34.4%),^6^ the mortality rate of COVID-19 is not as high, but the speed of transmission is faster, the infection is stronger, and the number of infected persons is higher. Therefore, we still need to be vigilant against this disease. We counted the number of days from the first symptom to death of 40 cases. The median survival time (in days) from onset to death was 13, the same as reported by Wang et al.^18^ Also, using single-factor analysis, we found that age was the key factor. Our study demonstrated that the time from the first symptom to death in the elderly (age > 65 y) was shorter than that of younger people (age ≤ 65 y), which is similar to that reported for MERS and SARS.^27,28^ On the other hand, other studies of patients with MERS found that patients with certain chronic diseases were more likely to die.^21-23^ However, our single-factor study did not find that these factors had statistical significance for the time. It may be that the sample size was too small. We suspect that people with these chronic diseases are more likely to become infected with COVID-19, but not to die. It also indirectly proves that this disease has a widespread, high infection rate, but low mortality compared with MERS and SARS. All in all, in the outbreak of COVID-19, we should pay attention to the health management and protection of the elderly.

Fortunately, academician Zhong Nanshan and his team from China released the guidelines for the prevention of COVID-19 among the elderly.^29^ The guidelines point out that the prevention of COVID-19 should be carried out according to age stratification, because the immunity of the elderly is relatively low. It is necessary to ensure the health of the elderly people. Also, the chronic diseases related to the elderly should be actively treated at the same time. We also call on everyone to pay special attention to the health management of the elderly, which is consistent with our conclusion.

There are several limitations to this study. The majority of cases are from Wuhan, Hubei Province, China. Second, only fatal cases were reported. It is preferable to add a considerable number of cases of survival. Finally, relevant laboratory test results and imaging data are lacking.

## CONCLUSION

In our study, we found that most of the deaths were elderly men with chronic fundamental diseases, and their time of COVID-19 progression to death was shorter. We also demonstrated that older men were more likely to be infected with COVID-19 and the risk of death is positively correlated with age. All in all, we still need to pay more attention to the elderly patients who are infected with the SARS-CoV-2 virus.
